# Supplementary material for: Enhancing Adhesion and Reducing Ohmic Contact through Nickel–Silicon Alloy Seed Layer in Electroplating Ni/Cu/Ag
Source: Materials (Basel). 2024 May 28;17(11):2610. doi: 10.3390/ma17112610 (PMC11173731; doi:10.3390/ma17112610)
Supplement: Supplementary file 1 [file materials-17-02610-s001.zip › materials-3002701-supplementary.pdf]

Supplementary Materials for

Enhancing Adhesion and Reducing Ohmic Contact through Nickel–Silicon Alloy Seed Layer in Electroplating Ni/Cu/Ag

Zhao Wang [1,2], Haixia Liu [1], Daming Chen [2], Zigang Wang [2], Kuiyi Wu [2], Guanggui Cheng [1], Yu Ding [1], Zhuohan Zhang [2,*], Yifeng Chen [2], Jifan Gao [2] and Jianning Ding [1,3,*]

[1] School of Materials Science and Engineering, Jiangsu University, Zhenjiang 212013, China
[2] State Key Lab of Photovoltaic Science and Technology, Trina Solar Co., Ltd., Changzhou 213031, China
[3] Institute of Technology for Carbon Neutralization, Yangzhou University, Yangzhou 225127, China
[*] Correspondence: zhao.wang02@trinasolar.com (Z.Z.); dingjn@yzu.edu.cn (J.D.)

Abstract: Due to the lower cost compared to screen-printed silver contacts, the Ni/Cu/Ag contacts formed by plating have been continuously studied as a potential metallization technology for solar cells. To address the adhesion issue of backside grid lines in electroplated n-Tunnel Oxide Passivating Contacts (n-TOPCon) solar cells and reduce ohmic contact, we propose a novel approach of adding a Ni/Si alloy seed layer between the Ni and Si layers. The metal nickel layer is deposited on the backside of the solar cells using electron beam evaporation, and excess nickel is removed by H_2SO_4:H_2O_2 etchant under annealing conditions of 300–425 °C to form a seed layer. The adhesion strength increased by more than 0.5 N mm^{-1} and the contact resistance dropped by 0.5 mΩ cm^2 in comparison to the traditional direct plating Ni/Cu/Ag method. This is because the resulting Ni/Si alloy has outstanding electrical conductivity, and the produced Ni/Si alloy has higher adhesion over direct contact between the nickel–silicon interface, as well as enhanced surface roughness. The results showed that at an annealing temperature of 375 °C, the main compound formed was NiSi, with a contact resistance of 1 mΩ cm^{-2} and a maximum gate line adhesion of 2.7 N mm^{-1}. This method proposes a new technical solution for cost reduction and efficiency improvement of n-TOPCon solar cells.

Keywords: Ni/Si alloy; annealing temperature; adhesion; plating; n-TOPCon solar cell

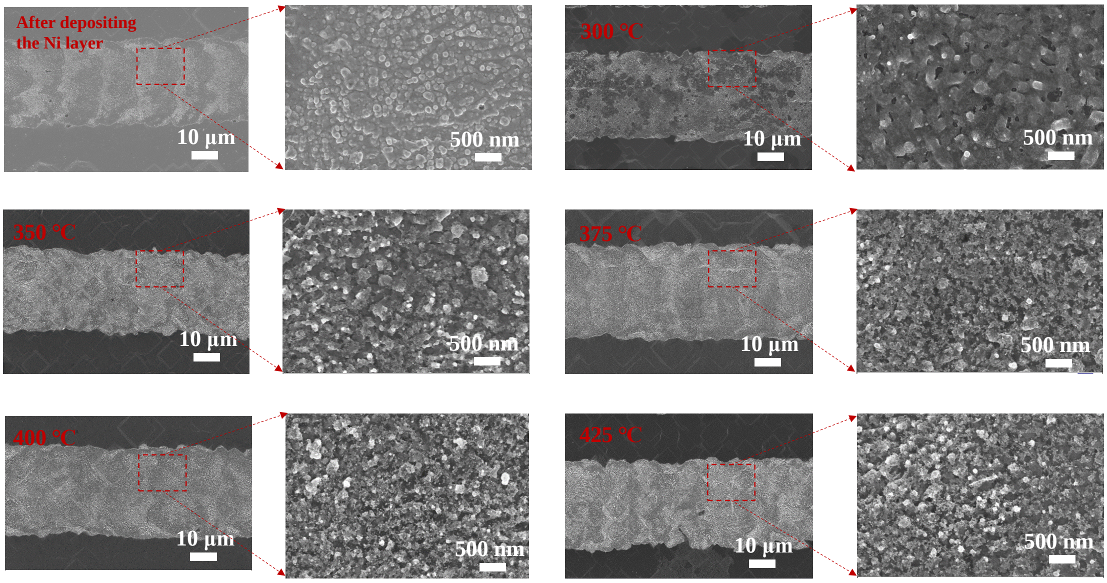

Figure S1: SEM images of samples at different annealing temperatures.

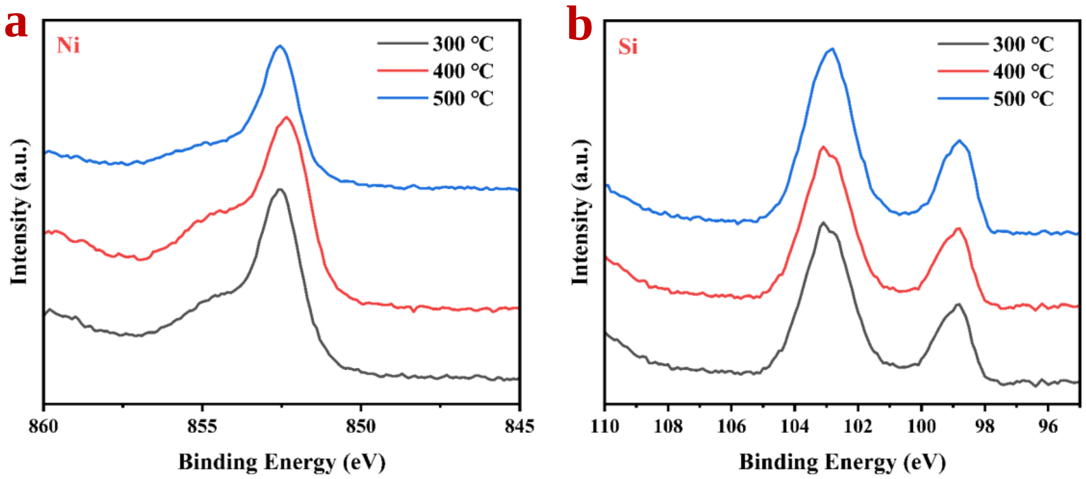

Figure S2: XPS diagram of samples at different annealing temperatures (a) Ni peaks; (b) Si peaks.
